# SARS-CoV-2 infection reduces human nasopharyngeal commensal microbiome with inclusion of pathobionts

**DOI:** 10.1038/s41598-021-03245-4

**Published:** 2021-12-15

**Authors:** M. Nazmul Hoque, Md. Murshed Hasan Sarkar, M. Shaminur Rahman, Shahina Akter, Tanjina Akhtar Banu, Barna Goswami, Iffat Jahan, M. Saddam Hossain, A. K. Mohammad Shamsuzzaman, Tasnim Nafisa, M. Maruf Ahmed Molla, Mahmuda Yeasmin, Asish Kumar Ghosh, Eshrar Osman, S. K. Saiful Alam, Mohammad Samir Uzzaman, Md Ahashan Habib, Abu Sayeed Mohammad Mahmud, Keith A. Crandall, Tofazzal Islam, Md. Salim Khan

**Affiliations:** 1 Department of Gynecology, Obstetrics and Reproductive Health, Bangabandhu Sheikh Mujibur Rahman Agricultural University (BSMRAU), Gazipur, 1706 Bangladesh; 2 Bangladesh Council of Scientific and Industrial Research (BCSIR), Dhanmondi, Dhaka, 1205 Bangladesh; 3 Department of Microbiology, Jashore University of Science Technology, Jashore, 7408 Bangladesh; 4 National Institute of Laboratory Medicine and Referral Center, Dhaka, 1207 Bangladesh; 5 SciTech Consulting and Solutions, Dhaka, 1213 Bangladesh; 6 Shaheed Tajuddin Ahmad Medical College, Gazipur, 1700 Bangladesh; 7 Computational Biology Institute and Department of Biostatistics and Bioinformatics, Milken Institute School of Public Health, The George Washington University, Washington, DC USA; 8 Institute of Biotechnology and Genetic Engineering (IBGE), BSMRAU, Gazipur, 1706 Bangladesh

**Keywords:** Computational biology and bioinformatics, Genetics, Microbiology, Molecular biology

## Abstract

The microbiota of the nasopharyngeal tract (NT) play a role in host immunity against respiratory infectious diseases. However, scant information is available on interactions of SARS-CoV-2 with the nasopharyngeal microbiome. This study characterizes the effects of SARS-CoV-2 infection on human nasopharyngeal microbiomes and their relevant metabolic functions. Twenty-two (n = 22) nasopharyngeal swab samples (including COVID-19 patients = 8, recovered humans = 7, and healthy people = 7) were collected, and underwent to RNAseq-based metagenomic investigation. Our RNAseq data mapped to 2281 bacterial species (including 1477, 919 and 676 in healthy, COVID-19 and recovered metagenomes, respectively) indicating a distinct microbiome dysbiosis. The COVID-19 and recovered samples included 67% and 77% opportunistic bacterial species, respectively compared to healthy controls. Notably, 79% commensal bacterial species found in healthy controls were not detected in COVID-19 and recovered people. Similar dysbiosis was also found in viral and archaeal fraction of the nasopharyngeal microbiomes. We also detected several altered metabolic pathways and functional genes in the progression and pathophysiology of COVID-19. The nasopharyngeal microbiome dysbiosis and their genomic features determined by our RNAseq analyses shed light on early interactions of SARS-CoV-2 with the nasopharyngeal resident microbiota that might be helpful for developing microbiome-based diagnostics and therapeutics for this novel pandemic disease.

## Introduction

Immediately after the first emergence of novel coronavirus SARS-CoV-2 in the city of Wuhan, China in December 2019, this fearsome pathogen has created a catastrophe known as COVID-19 spreading across 217 countries and/or territories of the globe as a pandemic^1,2^. SARS-CoV-2 emerged as one of the deadliest human pathogens in the last 100 years after the Spanish Flu in 1918–1920^3^. The virus primarily enters the human body mainly through the ACE2 and TMPRSS2 receptors, and nasal epithelial cells of the NT of the respiratory system^4^, and then gradually move towards the lung to initiate infection^5–7^. The pathophysiology of SARS-CoV-2 infections can be attributed to aberrant immune responses in clearing the virus^8,9^. Given the unequivocal association between viral and bacterial co-infections and respiratory disease severity, there is a pressing need to better understand how interactions of SARS-CoV-2 with the host microbiome in the respiratory tracts correlate with viral infections that facilitate opportunistic co-infections^10^.

Human microbiota play a crucial role in immunity and health of individual hosts. Any alterations of the diversity and population of the resident microbiota are associated with various chronic and acute human diseases^8,11^. In fact, the host‐microbiota symbiotic equilibrium is highly complex and associated with a number of intrinsic and environmental factors. Disruption of the homeostasis in composition of human microbiota leads to a state of “dysbiosis”^12^. Therefore, microbiome dysbiosis in the respiratory tract by the pathogenic respiratory virus can increase the mortality rate in patients^13,14^. Clinical trials and high throughput sequencing (metagenomic and RNAseq)-based investigations revealed the co-presence of diverse viruses, bacteria, archaea and fungi in the respiratory tracts of SARS-CoV-2 infected patients^15,16^. Recent studies of bronchoalveolar lavage fluid showed about 50% of the patients who died of COVID-19 had secondary bacterial infections^16,17^.

**Figure 1:**
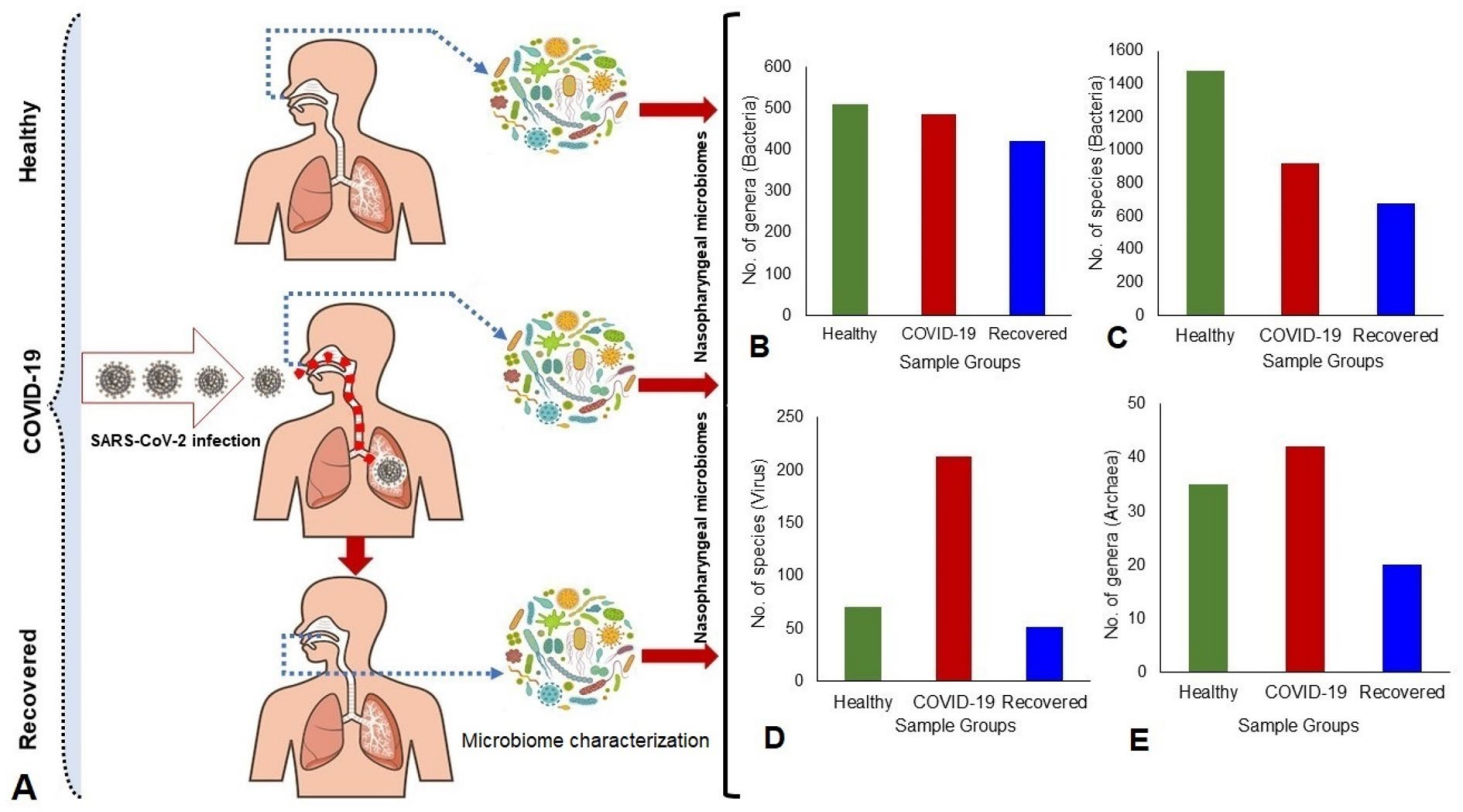
Experimental design and microbiome characterization. (**A**) Study sample groups: COVID-19, Recovered and Healthy humans. (**B**–**E**). Differences in microbiome (bacteria, viruses and archaea) composition and diversities across three metagenomes. The figure is generated using Microsoft PowerPoint, Excel and Adobe Illustrators.

Human health is the outcome of the complex interactions between the inhabiting microbiome and host^18^. The inhaled SARS-CoV-2 virus particle likely binds to epithelial cells in the nasal cavity, replicates and then migrates down the respiratory tract along the conducting airways which triggers a robust innate immune response^4,5^. Therefore, it can assume that during this propagation, migration and immune response, the resident microbiomes in the respiratory airways could be altered or changed, and inclusion of some of the pathobionts might aggravate the progression and lethality of the disease caused by SARS-CoV-2. Although several lines of evidence suggest that development of COVID-19 disease modulates the population and diversity of resident commensal microbiota of humans, little is known about the outcome of the interactions of SARS-CoV-2 with nasal commensal microbiome which is thought to be critical for transmission, modulation, and progression of COVID-19^19,20^. It is important to understand the interactions of SARS-CoV-2 on the composition and diversity of microbiomes in the NT of COVID-19 and recovered patients compared to the healthy individuals. To shed light on the effects and consequences of SARS-CoV-2 infection on the NT microbiome, we conducted a high throughput RNAseq analysis of the nasopharyngeal swabs of randomly selected healthy humans, COVID-19 and recovered patients. Furthermore, we conducted a functional analysis to identify potential biological mechanisms linking the shift of microbiome, SARS-CoV-2 genomic diversity and host disease in COVID-19, recovered and healthy states (Fig. 1). This report for the first time demonstrates the association of microbiome diversity and composition (Fig. 1), and their concomitant genomic features in the nasal cavity of COVID-19 and recovered patients compared to healthy humans, and discusses the role of the altered microbiome in the pathophysiology of the SARS-CoV-2 infections.

## Results

The first step in managing COVID-19 is the rapid and accurate detection of SARS-CoV-2 by quantitative reverse transcription–polymerase chain reaction (RT–qPCR). In this study, patients were diagnosed positive for COVID-19 on an average 4.7 days (range 2–9 days) after the onset of clinical signs, and these patients tested negative for SARS-CoV-2 (Recovered) on an average 17.5 days (range 11–32 days) after the initial COVID-19 confirmatory diagnosis (Table S1). The Healthy control subjects however did not show any signs and symptoms of respiratory illness.

### SARS-CoV-2 infection modulates the community composition and diversity of nasopharyngeal microbiomes

**Figure 2:**
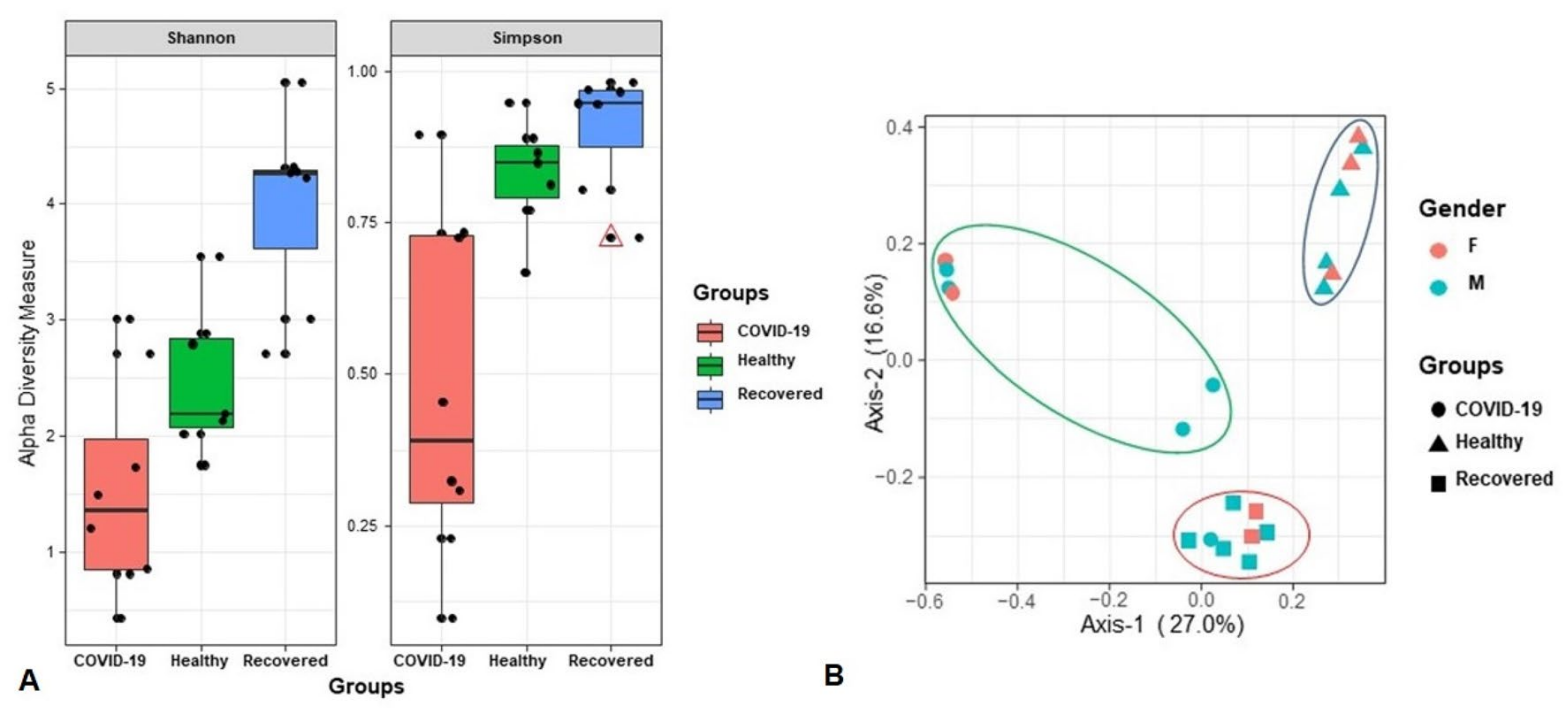
Differences in microbiome diversity and community structure in COVID-19, Recovered and Healthy nasopharyngeal sample metagenomes. (**A**) Box plots showing higher diversity in the microbial niche of recovered (p = 0.0065; Kruskal–Wallis test) followed by healthy (p = 0.0041; Kruskal–Wallis test) and COVID-19 (p = 0.0105; Kruskal–Wallis test) samples in both Shannon and Simpson estimated alpha diversity calculation. The line inside the box represents the median, and outliers as well as individual sample values are shown as dots. (**B**) Principal coordinates analysis (PCoA) measured on the Bray–Curtis distance method separated samples by microbial population structure. Each color represents an individual study people either as male (M) or female (F). The sample groups are denoted using different shapes like circular: COVID-19, rectangle: Recovered, and triangle: Healthy controls. Statistical analysis using Kruskal–Wallis tests showed significant microbial diversity variations across the three metagenomes (p = 0.0059, Kruskal–Wallis test). Data were processed through Phyloseq (https://www.bioconductor.org/packages/release/bioc/html/phyloseq.html) R package, visualized by using ggplot2 (https://cran.r-project.org/web/packages/ggplot2/index.html).

To shed light on the effects of SARS-CoV-2 infections in the diversity and composition of human nasal microbiome, we analyzed RNAseq data of nasopharyngeal samples of randomly selected healthy human, COVID-19 and recovered patients (Fig. 1). The alpha-diversity (within sample diversity through Shannon and Simpson indices) assessment revealed significant differences in microbial species richness across three metagenomes regardless of the method used to tabulate microbial abundances i.e., either IDseq or MG-RAST (MR), showing higher diversity in the microbial niche of Recovered (p = 0.0065; Kruskal–Wallis test) followed by Healthy (p = 0.0041; Kruskal–Wallis test) and COVID-19 (p = 0.0105; Kruskal–Wallis test) samples (Fig. 2A). The beta diversity (between sample or metagenome diversity) through principal coordinate analysis (PCoA), as measured on the Bray–Curtis distance method using IDseq and MR data, showed distinct discrimination across the metagenomes and separated samples by microbial population structure (Fig. 2B). Therefore, significant variation in microbiome diversity and composition across these three metagenomes (p = 0.0059, Kruskal–Wallis test) was evident. The diversity of microbiomes between samples (PCoA) however did not vary significantly (p = 0.651, Kruskal–Wallis rank sum test) according to the gender (male or female) of the study population (Fig. 2B).

**Figure 3:**
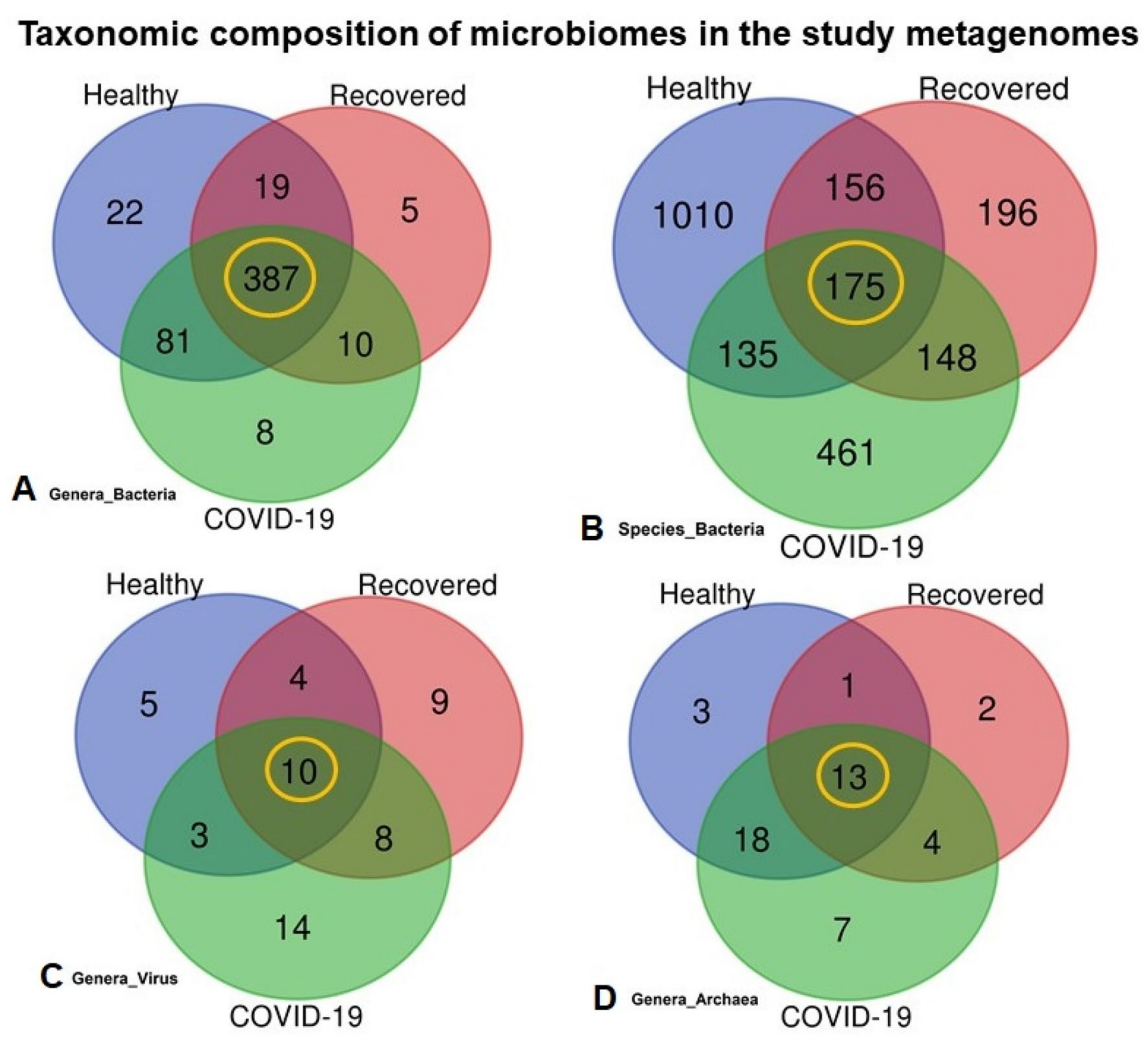
Taxonomic composition of the nasopharyngeal microbiomes of COVID-19 patients, Recovered people and Healthy controls. Venn diagrams representing the core unique and shared microbiomes in COVID-19, Recovered and Healthy samples. (**A**) Venn diagram showing unique and shared bacterial genera. Out of 532 genera detected, only 387 genera (highlighted in yellow) were found to be shared across three sample categories. (**B**) Venn diagram comparison of unique and shared bacterial species where only 175 (highlighted in yellow) species shared in the study metagenomes, and 461, 196 and 1,010 species had sole association with COVID-19, Recovered and Healthy samples, respectively. (**C**) Venn diagrams representing unique and shared viral genera identified in three metagenomes. Of the detected viral genera (n = 53), 14, 9 and 7 genera had unique association with COVID-19, Recovered and Healthy samples, respectively, and 10 genera (highlighted in yellow) were found to be shared across three metagenomes. (**D**) Venn diagrams showing unique and shared archaeal genera in COVID-19, Recovered and Healthy nasopharyngeal samples. A total of forty-eight archaeal genera (n = 48) was detected, of which 7, 2 and 3 genera had unique association with COVID-19, Recovered and Healthy samples, respectively, and 13 genera (highlighted in yellow) were found to be shared across three metagenomes. More information on the taxonomic result is also available in Data S1. Venn diagrams are generated through an online tool used for Bioinformatics and Evolutionary Genomics (http://bioinformatics.psb.ugent.be/webtools/Venn/).

Microbiome composition at the domain level was numerically dominated by bacteria, with a relative abundance of 81.58%, followed by viruses (18.22%) and archaea (0.20%) (Fig. 1, Data S1). The unique and shared distribution of microbes found in the three participant groups are represented by comprehensive Venn diagrams (Fig. 3). In this study, we detected 532 bacterial genera, including 486, 421 and 509 in COVID-19, Recovered and Healthy nasopharyngeal samples, respectively, and of them, 72.74% genera were common in three sample groups (Fig. 3A, Data S1). Notably, we detected 2281 bacterial species through IDseq analysis, of which 919, 675 and 1476 species were found in COVID-19, Recovered, and Healthy samples, respectively (Fig. 3B, Data S1). However, compared to COVID-19 and Recovered samples, the Healthy samples had unique or sole association of 1010 (68.43%) bacterial species which underwent dysbiosis during the pathogenesis and subsequent recovery phase of COVID-19 (Data S2). Of the identified bacterial species, 50.16% and 29.04% had sole association in COVID-19 and Recovered samples, respectively indicating their opportunistic inclusion in COVID-19 patients and re-establishment beneficial commensal flora with the recovery of SARS-CoV-2 infections (Fig. 3B, Table 1, Data S2).

**Table 1:** Top abundant commensal and pathogenic (mostly opportunistic) nasopharyngeal bacteria identified in current and previous studies.

Bacterial species	Sample groups			Virulence status		Previous reports
	Healthy	COVID-19	Recovered	Commensal	Pathogenic	
*Pseudomonas* sp. LPH1	** + **	**−**	** + **	Y	Y	^48,49,50^
*Rheinheimera* sp. D18	** + **	** + **	** + **	NR	Y	^51^
*Pseudomonas mendocina*	** + **	** + **	** + **	Y	Y	^48,49,50^
*Brevundimonas* sp. Bb-A	** + **	**−**	** + **	Y	NR	^50,52^
*Enterobacter hormaechei*	** + **	** + **	** + **	NR	Y	^53,54^
*Pseudomonas oleovorans*	** + **	**−**	** + **	Y	Y	^50,55^
*Klebsiella pneumoniae*	** + **	** + **	** + **	NR	Y	^56,57^
*Orbus* sp. IPMB12	** + **	** + **	**−**	NR	NR	NR
*Rheinheimera* sp. LHK132	** + **	** + **	** + **	NR	Y	^51^
*Pseudomonas* sp. phDV1	** + **	**−**	**−**	Y	Y	^48^
*Empedobacter brevis*	** + **	** + **	** + **	NR	Y	^58^
*Brevundimonas* sp. DS20	** + **	**−**	** + **	NR	Y	^52^
*Streptococcus salivarius*	**−**	** + **	** + **	Y	NR	^59,60^
*Streptococcus mitis*	**−**	** + **	** + **	Y	Y	^50,61,62,63^
*Neisseria subflava*	**−**	** + **	** + **	Y	NR	^50,64,65^
*Veillonella dispar*	**−**	** + **	** + **	Y	Y	^50,63,66^
*Veillonella parvula*	**−**	** + **	** + **	Y	Y	^50,63,66^
*Prevotella melaninogenica*	**−**	** + **	** + **	Y	Y	^50,63,66^
*Streptococcus parasanguinis*	**−**	** + **	**−**	Y	Y	^50,60,62,67^
*Streptococcus* sp. LPB0220	**−**	** + **	**−**	Y	Y	^50,60,63,67^
*Haemophilus parainfluenzae*	** + **	** + **	** + **	Y	Y	^50,63,68,69^
*Streptococcus pneumoniae*	** + **	** + **	** + **	NR	Y	^50,63,67,70^
*Pseudomonas stutzeri*	** + **	** + **	** + **	Y	Y	^48,49,50^
*Staphylococcus capitis*	**−**	** + **	** + **	NR	Y	^62,71^
*Staphylococcus epidermidis*	** + **	** + **	** + **	Y	Y	^15,62,72^
*Cupriavidus metallidurans*	** + **	**−**	** + **	NR	Y	^73,74^
*Moraxella osloensis*	** + **	** + **	** + **	NR	Y	^57,62^
*Acinetobacter indicus*	** + **	** + **	** + **	Y	Y	^75^
*Escherichia coli*	** + **	** + **	** + **	Y	Y	^63,76^
*Sphingobacterium* sp. G1-14	** + **	** + **	** + **	NR	Y	^77^
*Acinetobacter junii*	** + **	** + **	** + **	NR	Y	^75^
*Ralstonia pickettii*	**−**	** + **	** + **	NR	Y	^78^

Here: ‘+’ refers to particular species present in the corresponding sample group while ‘−’ refers to the absence, Y yes, either commensal or opportunistic, NR not reported.

The MR pipeline detected 53 viral and 48 archaeal genera in three metagenomes, and of them, 18.87% viral (Fig. 3C), and 27.08% archaeal (Fig. 3D) genera were found to be shared in the metagenomes of COVID-19, Recovered and Healthy samples. By comparing these genera across the sample categories, we identified 35, 31 and 22 viral (Fig. 3C), and 42, 20 and 35 archaeal (Fig. 3D) genera in COVID-19, Recovered and Healthy-swabs, respectively (Table S2). We found that 22.73% and 29.03% viral (Fig. 3B), and 8.57%, and 10.0% archaeal genera in healthy and recovered samples, respectively shared with those of COVID-19 samples. The COVID-19 patients had sole association of 40.0% viral, and 16.67% archaeal genera (Fig. 3C,D, Table S2, Data S1). Besides, 213, 52 and 71 viral species (bacteriophages mostly) were identified in COVID-19, Recovered and Healthy metagenomes, respectively. Among the detected viral species, 97.65% and 98.07% had sole association with COVID-19 and recovered samples, respectively (Data S1).

### SARS-CoV-2 infection reduces the diversity and composition of nasopharyngeal commensal bacteria

The present microbiome study demonstrated that both the composition and the relative abundances of bacterial taxa at the phylum, order, family, genus, and species-level differed significantly (p = 0.031, Kruskal Wallis test) among COVID-19, Recovered and Healthy controls (Fig. 1A,B). *Firmicutes* was found to be the most predominant bacterial phylum in COVID-19 and Recovered metagenomes with a relative abundance of 76.31% and 39.28%, respectively (Data S1). The other predominant bacterial phyla were *Bacteroidetes* (7.07%), *Proteobacteria* (6.85%), *Fusobacteria* (5.37%), *Actinobacteria* (1.45%), and *Cyanobacteria* (1.23%) in COVID-19 metagenome, and *Proteobacteria* (27.42%), *Actinobacteria* (16.61%), *Bacteroidetes* (12.86%), and *Cyanobacteria* (1.71%) in Recovered metagenome (Data S1). On the other hand, *Proteobacteria* (50.19%), *Bacteroidetes* (41.60%), and *Firmicutes* (6.42%) were the most abundant phyla in Healthy control samples (Data S1).

**Figure 4:**
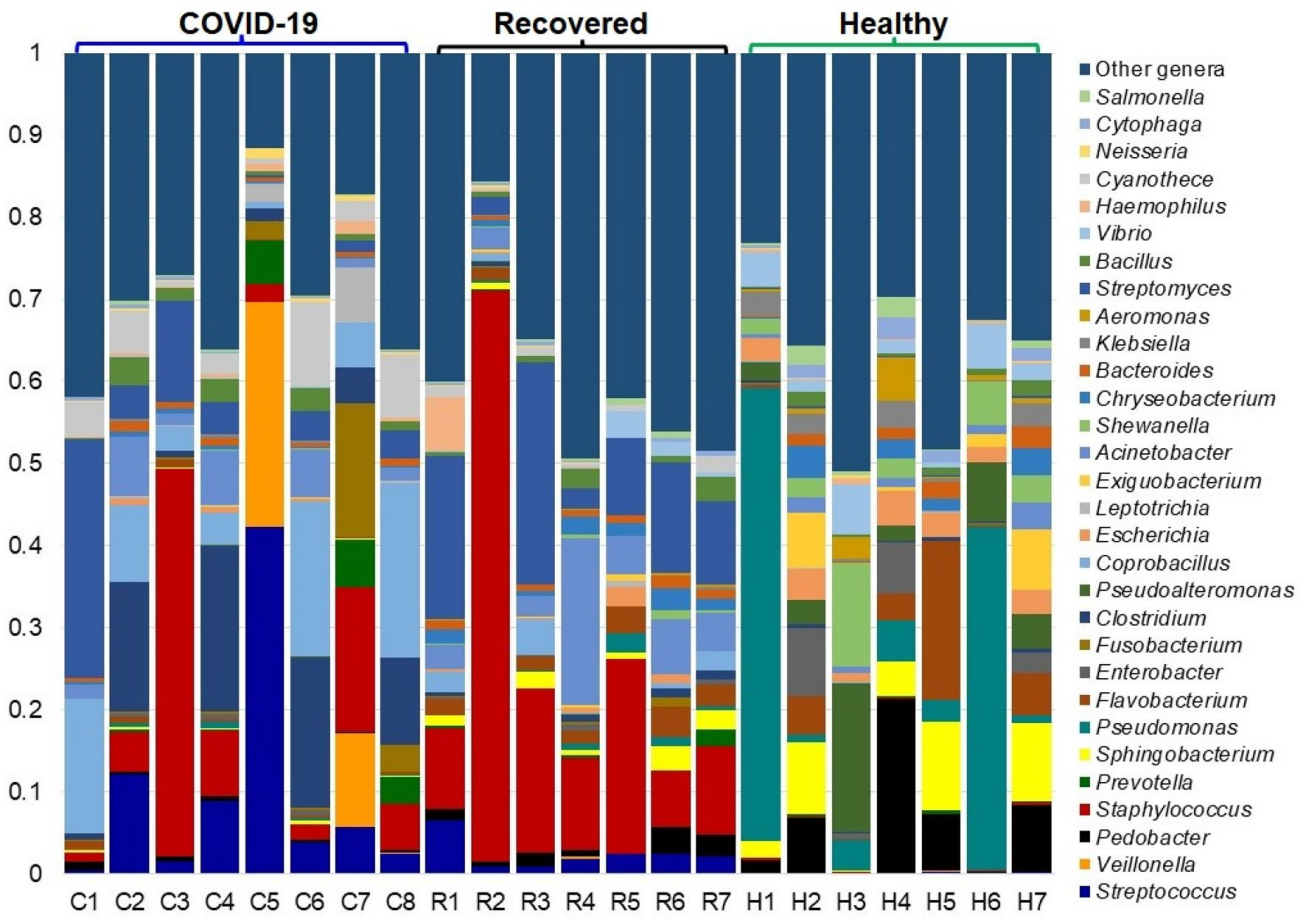
The genus-level taxonomic profile of bacteria in COVID-19 (C1–C8), Recovered (R1–R7) and Healthy (H1–H7) nasopharyngeal samples. The relative abundance of 30 most abundant bacterial genera are sorted from bottom to top by their deceasing proportion, with the remaining genera keeping as ‘Other genera’. Each stacked bar plot represents the abundance of bacterial genera in each sample of the corresponding category. Notable differences in bacterial populations are those where the taxon is abundant in COVID-19 and Recovered samples, and effectively undetected in the Healthy controls. The distribution and relative abundance of the bacterial genera in the study metagenomes are also available in Data S1. The bar plot is generated using Microsoft Excel program.

We found significant differences (p = 0.021, Kruskal–Wallis test) in the diversity and relative abundance of the bacteria at the genus level. The individual and inter-individual microbiome variability showed that Healthy individuals had higher number of bacterial genera (average 318.57/person) compared to COVID-19 (234.50/patient) and Recovered (156.29/person) patients (Fig. S1A). However, the bacterial genera detected in the metagenome COVID-19 patient mapped to the highest number of reads per genus (476.95 reads/genus) compared to the Healthy (278.89 reads/genus) and Recovered (37.36 reads/genus) individuals (Fig. 1A, Fig. S1B). In COVID-19 metagenome, *Streptococcus* was the most abundant bacterial pathogen with a relative abundance of 37.16% followed by *Veillonella* (24.25%), *Prevotella* (4.97%), *Staphylococcus* (3.49%), *Fusobacterium* (2.89%), *Clostridium* (2.59%), *Leptotrichia* (2.26%), and *Coprobacillus* (2.24%) (Fig. 4, Data S1). Likewise, top abundant bacterial genera in the metagenome of Recovered individuals were *Staphylococcus* (28.82%), *Streptomyces* (9.11%), *Acinetobacter* (8.96%), *Corynebacterium* (5.18%), *Streptococcus* (2.48%), and *Helicobacter* (2.42%) (Fig. 4, Data S1). Conversely, the Healthy control metagenome was predominated by *Pedobacter* (11.69%), *Sphingobacterium* (6.35%), *Pseudomonas* (5.03%), *Enterobacter* (4.79%), *Flavobacterium* (4.62%), *Pseudoalteromonas* (3.82%), *Escherichia* (3.50%), *Exiguobacterium* (3.38%), *Shewanella* (3.16%), *Chryseobacterium* (2.58%), *Aeromonas* (2.52%), *Klebsiella* (2.50%), and *Vibrio* (2.03%). The rest of the genera detected in these metagenomes had relatively lower abundances (< 2.0%) (Fig. 4, Data S1).

**Figure 5:**
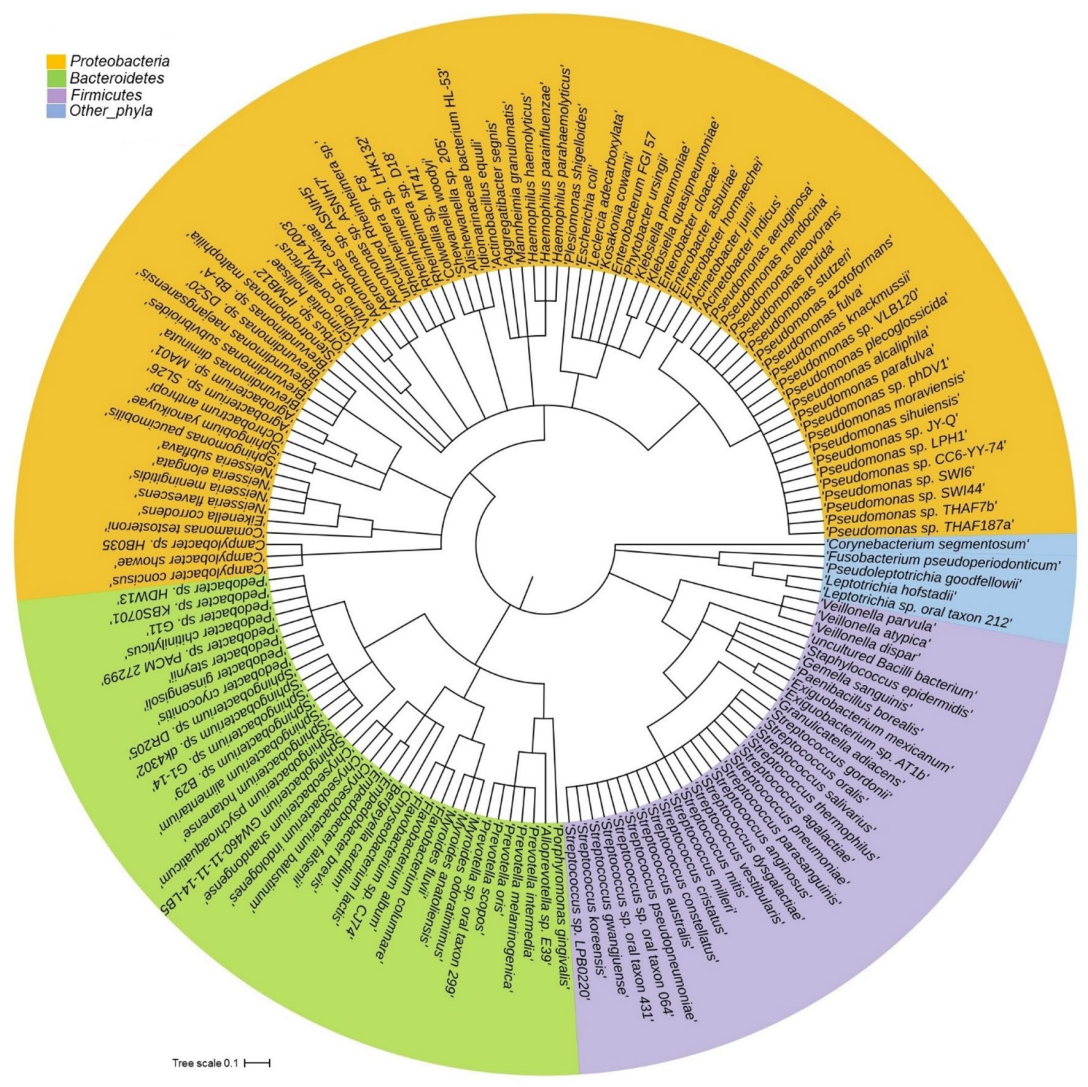
The species-level taxonomic representation of microbiome in COVID-19, Recovered and Healthy metagenomes. Taxonomic dendrogram in the midpoint rooted phylogenetic tree was generated with top abundant 200 species of bacteria found in three metagenomes. The tree was built based on the maximum likelihood method in Clustal W (https://www.ebi.ac.uk/Tools/msa/clustalo/) and displayed with iTOL (interactive Tree Of Life; https://itol.embl.de/). Color ranges identify different species within the tree. Species are color-coded by different phyla of bacteria present in > 90% of samples. The species-level information of the phylogenetic tree is also available in Data S1.

We further investigated the species-level differences of microbial communities across these three metagenomes through IDseq analysis, which revealed significant variations (p = 0.011, Kruskal–Wallis test) in microbiome composition, diversity and relative abundances (Fig. 5, Table 1, Data S1, S2). The COVID-19 metagenome was dominated by 73 species (7.94%) of *Streptococcus* genus while *Clostridium*, *Chrysobacterium*, *Paenibacillus*, *Neissaria*, *Acinetobacter*, *Staphylococcus*, and *Corynebacterium* genera were represented by 38, 26, 25, 23, 22, 22 and 17 different species, respectively. Similarly, the bacteriome of the Recovered people was dominated by different species of *Corynebacterium* (39), *Acinetobacter* (36), *Chryseobacterium* (30), *Sphingobacterium* (26), *Staphylococcus* (24), *Pseudomonas* (18), and *Flavobacterium* (16) (Fig. 5, Table 1, Data S1, S2). In contrast, the bacteriome of the Healthy controls was predominantly represented by 145 species (9.82%) of *Pseudomonas* genus followed by 49, 41, 38, 38, 36, 36, 33, and 26 species of *Vibrio*, *Shewanella*, *Acinetobacter*, *Flavobacterium*, *Chryseobacterium*, *Enterobacter*, *Pseudoalteromonas*, and *Sphingobacterium* genera, respectively (Fig. 5, Table 1). Remarkably, 68.43% (1010/1476) bacterial species were solely associated with the Healthy nasopharyngeal samples, and not detected in SARS-CoV-2 infected (COVID-19) and Recovered humans. Among these depleted commensal species *Pseudomonas* sp. LPH1 (25.32%), *Brevundimonas* sp. Bb-A (4.85%), *P. oleovorans* (3.25%), *Pseudomonas* sp. phDV1 (1.75%) and *Brevundimonas* sp. DS20 (1.38%) were top abundant (Fig. 5, Table 1, Data S1, S2). Conversely, the COVID-19 samples had sole association of 461 opportunistic bacterial species, and of them, *Streptococcus salivarius* K12 (19.13%), *S. mitis* (18.13%), *Neisseria subflava* (13.77%), *Veillonella dispar* (11.03%), *Acinetobacter junii 64.*5 (3.31%), *V. parvula* (2.35%), *Prevotella melaninogenica* (2.26%), *S. parasanguinis* (2.22%), *Streptococcus* sp. LPB0220 (1.98%), *N. flavescens* (1.26%), and *V. atypica* (1.11%) were top abundant (Fig. 5, Table 2, Data S2). Likewise, the Recovered humans of COVID-19 had sole association of 196 species with comparatively higher relative abundances of *Pseudomonas stutzeri* DSM 4166 (8.48%), *Staphylococcus capitis* (5.89%), *S. epidermidis* RP62A (5.13%), *P. mendocina* NK-01 (3.12%), *Moraxella osloensis* A1920 (2.60%), *A. indicus* A648 (2.57%), *Escherichia coli* (2.41%), *Sphingobacterium* sp. G1-14 (1.96%), *A. junii* 64.5 (1.85%), *S. pneumoniae* (1.60%), *Ralstonia pickettii* (1.55%), *Micrococcus luteus* (1.46%), *Rheinheimera* sp. D18 (1.33%), *Corynebacterium segmentosum* (1.26%), *Elizabethkingia anopheles* (1.25%), and *Cutibacterium acnes* (1.08%). The rest of the species in these metagenomes had relatively lower (< 1.0%) abundances (Fig. 5, Table 1, Data S2).

**Table 2:** Top abundant opportunistic bacterial species in COVID-19 and Recovered patients (not detected in Healthy controls).

Phylum	Species	Relative abundance (%)	
		COVID-19	Recovered
*Firmicutes*	*Streptococcus salivarius*	19.13	0.03
*Firmicutes*	*Streptococcus mitis*	18.13	0.11
*Proteobacteria*	*Neisseria subflava*	13.77	0.06
*Firmicutes*	*Veillonella dispar*	11.03	0.01
*Proteobacteria*	*Acinetobacter junii*	3.31	1.85
*Firmicutes*	*Veillonella parvula*	2.35	0.05
*Firmicutes*	*Prevotella melaninogenica*	2.26	0.08
*Firmicutes*	*Streptococcus parasanguinis*	2.22	0.0
*Firmicutes*	*Streptococcus *sp*. LPB0220*	1.98	0.02
*Proteobacteria*	*Neisseria flavescens*	1.26	0.0
*Firmicutes*	*Veillonella atypica*	1.10	0.02
*Proteobacteria*	*Pseudomonas stutzeri*	0.01	8.48
*Firmicutes*	*Staphylococcus capitis*	0.005	5.89
*Firmicutes*	*Staphylococcus epidermidis*	0.20	5.13
*Proteobacteria*	*Pseudomonas mendocina*	0.15	3.12
*Proteobacteria*	*Moraxella osloensis*	0.13	2.60
*Proteobacteria*	*Acinetobacter indicus*	0.05	2.57
*Proteobacteria*	*Escherichia coli*	0.12	2.41
*Bacteroidetes*	*Sphingobacterium *sp*. G1–14*	0.043	1.96
*Firmicutes*	*Streptococcus pneumoniae*	0.005	1.60
*Proteobacteria*	*Ralstonia pickettii*	0.002	1.55
*Actinobacteria*	*Micrococcus luteus*	0.035	1.46

### SARS-CoV-2 infection associated dysbiosis of viruses and archaea in the nasopharyngeal tract

**Figure 6:**
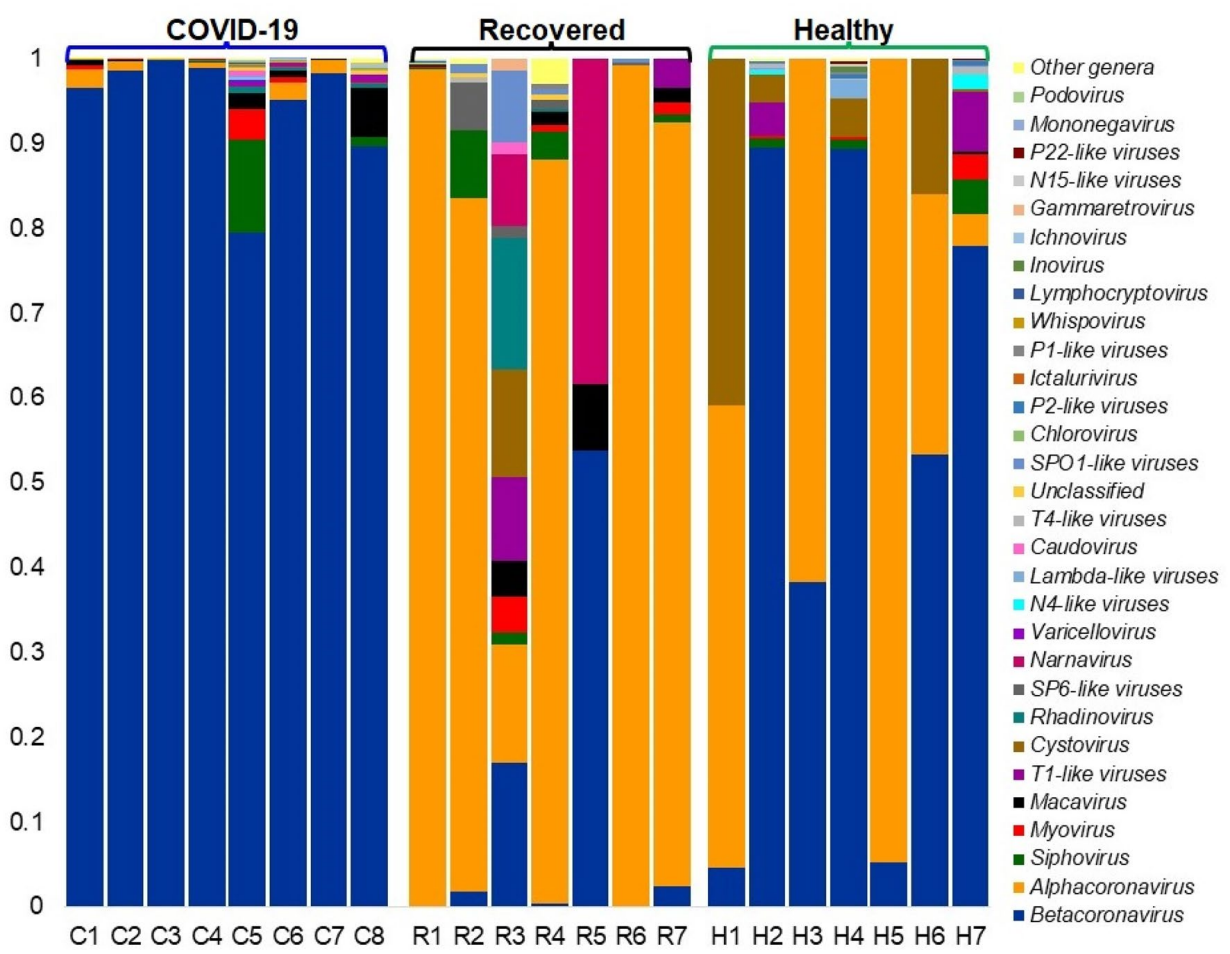
The genus-level taxonomic profile of virus in COVID-19 (C1–C8), Recovered (R1–R7) and Healthy (H1–H7) nasopharyngeal samples. Stacked bar plots showing the relative abundance and distribution of the 30 most top abundant viral genera, with ranks ordered from bottom to top by their decreasing proportion, with the remaining genera keeping as ‘Other genera’. Each stacked bar plot represents the abundance of viral genera in each sample of the corresponding category. Notable differences in viral populations are those where the taxon is abundant in COVID-19 and Recovered samples, and effectively undetected in the Healthy controls. The distribution and relative abundance of the archaeal genera in the study metagenomes are also available in Data S1. The bar plot is generated using Microsoft Excel program.

The COVID-19 patients and Recovered humans had higher number of viral genera and/or species compared to Healthy controls. In this study, *Betacoronavirus* was found as the top abundant viral genus in COVID-19 and Healthy samples with a relative abundance of 97.95% and 83.57%, respectively. In addition, other predominating viral genera in COVID-19 samples were *Alphacoronavirus* (0.90%) and *Siphovirus* (0.54%) while *Alphacoronavirus* (3.54%), *T1-like viruses* (3.25%), *Cystovirus* (3.23%), *Siphovirus* (1.91%), *Myovirus* (1.09%), *Lambda-like viruses* (0.78%), and *N4-like viruses* (0.72%) (Fig. 6, Data S1). Conversely, the Recovered samples were mostly dominated by *Alphacoronavirus* (92.54%) followed by *Betacoronavirus* (1.23%), *Siphovirus* (1.16%), *T1-like viruses* (0.81%), *Macavirus* (0.67%), and *SP6-like viruses* (0.56%) (Fig. 6, Data S1). Most of these viral genera had lower relative abundances in Healthy controls. In this study, the SARS-CoV-2 was the predominantly abundant viral strain in COVID-19 patients (99.44%) and Healthy controls (84.81%), whereas the *Alphacoronavirus* Human coronavirus NL63 (HCoV-NL63) was the most abundant (90.89%) viral strain in Recovered humans nasopharyngeal samples. The rest of the viral strains in three metagenomes were mostly different strains of bacterial phages. Remarkably, the nasopharyngeal samples from the COVID-19 patients were mostly enriched with different strains of *Streptococcal* phages besides the SARS-CoV-2. Similarly, *Caudovirales* phage, *Rheinheimera* virus Barba19A, *Bacillus* phage Chotacabras, *Staphylococcus* phage StB12, *Lactobacillus* virus Semele, *Propionibacterium* phage pa28, *Staphylococcus* virus SEP9, *Staphylococcus* phage CNPx, *Propionibacterium* virus Stormborn etc. were identified as the dominating viral strains in Recovered humans nasopharyngeal samples in addition to HCoV-NL63 (Data S1). Despite the predominant abundance of SARS-CoV-2, *Pseudomonas* virus phi12 (13.45%), and different strains of *Klebsiella* phages remained as the subsidiary viral strains in Healthy controls (Data S1).

**Figure 7:**
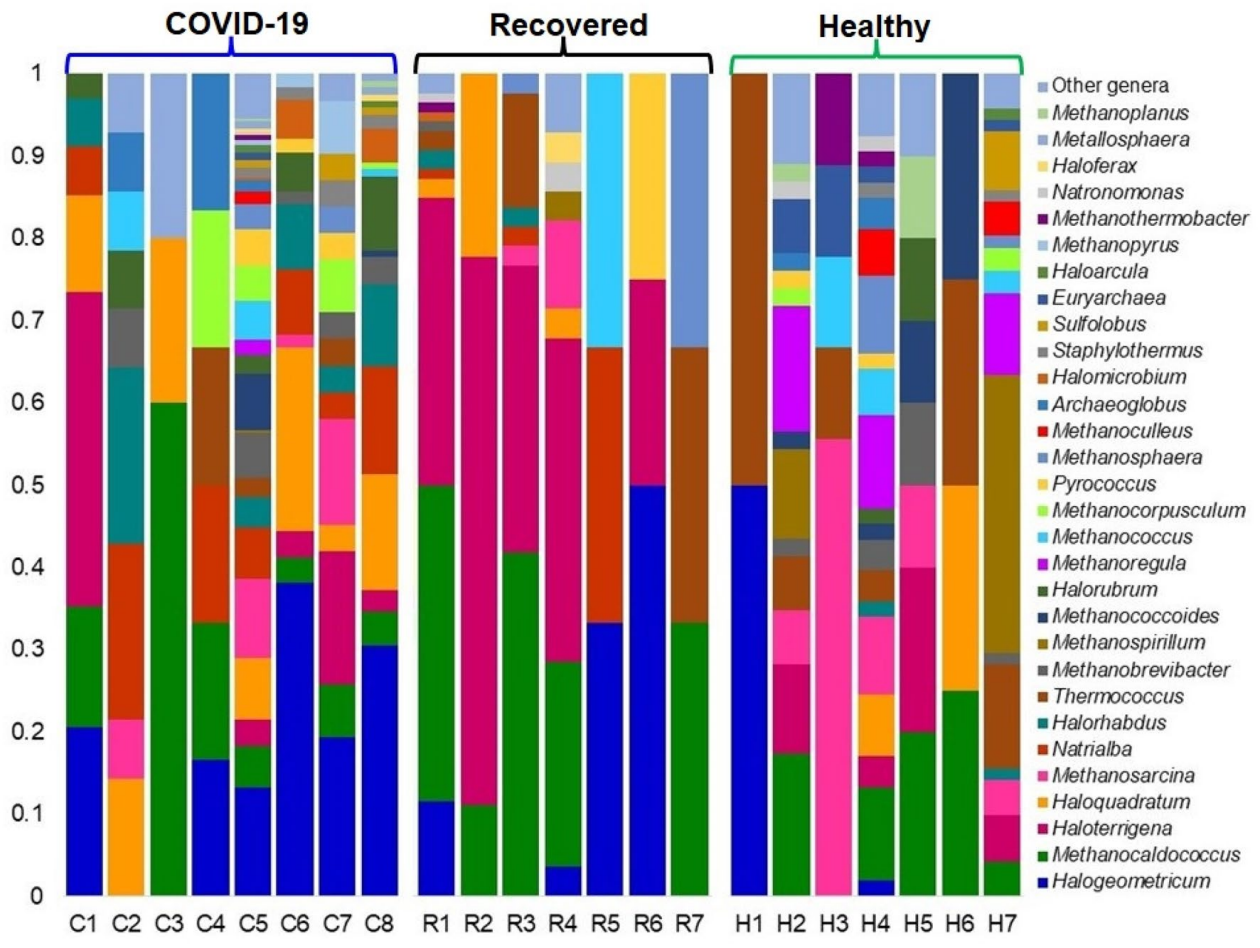
The genus-level taxonomic profile of archaea in COVID-19 (C1–C8), Recovered (R1–R7) and Healthy (H1–H7) nasopharyngeal samples. Stacked bar plots showing the relative abundance and distribution of the 30 most top abundant archaeal genera, with ranks ordered from bottom to top by their decreasing proportion, with the remaining genera keeping as ‘Other genera’. Each stacked bar plot represents the abundance of archaeal genera in each sample of the corresponding category. Notable differences in archaeal populations are those where the taxon is abundant in COVID-19 and Recovered samples, and effectively undetected in the Healthy controls. The distribution and relative abundance of the archaeal genera in the study metagenomes are also available in Data S1. The bar plot is generated using Microsoft Excel program.

Similar dysbiosis was also evident in the archaeal fraction of the microbiomes. For instance, the top abundant archaeal genera in COVID-19 samples were *Halogeometricum* (19.57%), *Haloquadratum* (10.53%), *Natrialba* (8.06%), *Methanosarcina* (6.25%), *Halorhabdus* (5.76%), *Methanocaldococcus* (5.76%), *Haloterrigena* (5.59%), *Methanobrevibacter* (4.11%), *Halorubrum* (3.95%), *Methanococcoides* (3.95%), *Methanococcus* (2.96%), and *Methanocorpusculum* (2.96%). Correspondingly, *Haloterrigena* (36.47%), *Methanocaldococcus* (35.29%), *Halogeometricum* (6.47%), *Thermococcus* (5.29%), *Haloquadratum* (2.94%), *Methanosarcina* (2.35%), and *Pyrococcus* (2.80%) were the predominating archaeal genera in the Recovered patient’s samples (Fig. 7, Data S1). The Healthy control samples, however had higher relative abundances of *Methanospirillum* (15.34%), *Methanoregula* (10.58%), *Methanocaldococcus* (10.05%), *Methanosarcina* (9.00%), *Thermococcus* (7.94%), *Haloterrigena* (6.88%), *Methanococcus* (3.18%), *Methanoculleus* (3.18%), *Methanosphaera* (3.18%), *Euryarchaeota* (3.18%), *Methanobrevibacter* (2.65%), *Sulfolobus* (2.65%), and *Haloquadratum* (2.12%) genera. The rest of the archaeal genera in the study metagenomes had lower relative abundances (< 2.0%) (Fig. 7, Data S1). Notably, among the unique archaeal genera detected in Healthy control samples, *Methanopyrus* (0.82%) and *Metallosphaera* (0.66%) were predominantly abundant genera (Data S2). Similarly, the Recovered samples also had sole association of 2 (10.0%) archaeal genera, and the relative abundances of these genera remained much lower (< 1.0%) in both metagenomes (Data S2).

### SARS-CoV-2 infection associated changes in genomic potentials of the microbiomes

**Figure 8:**
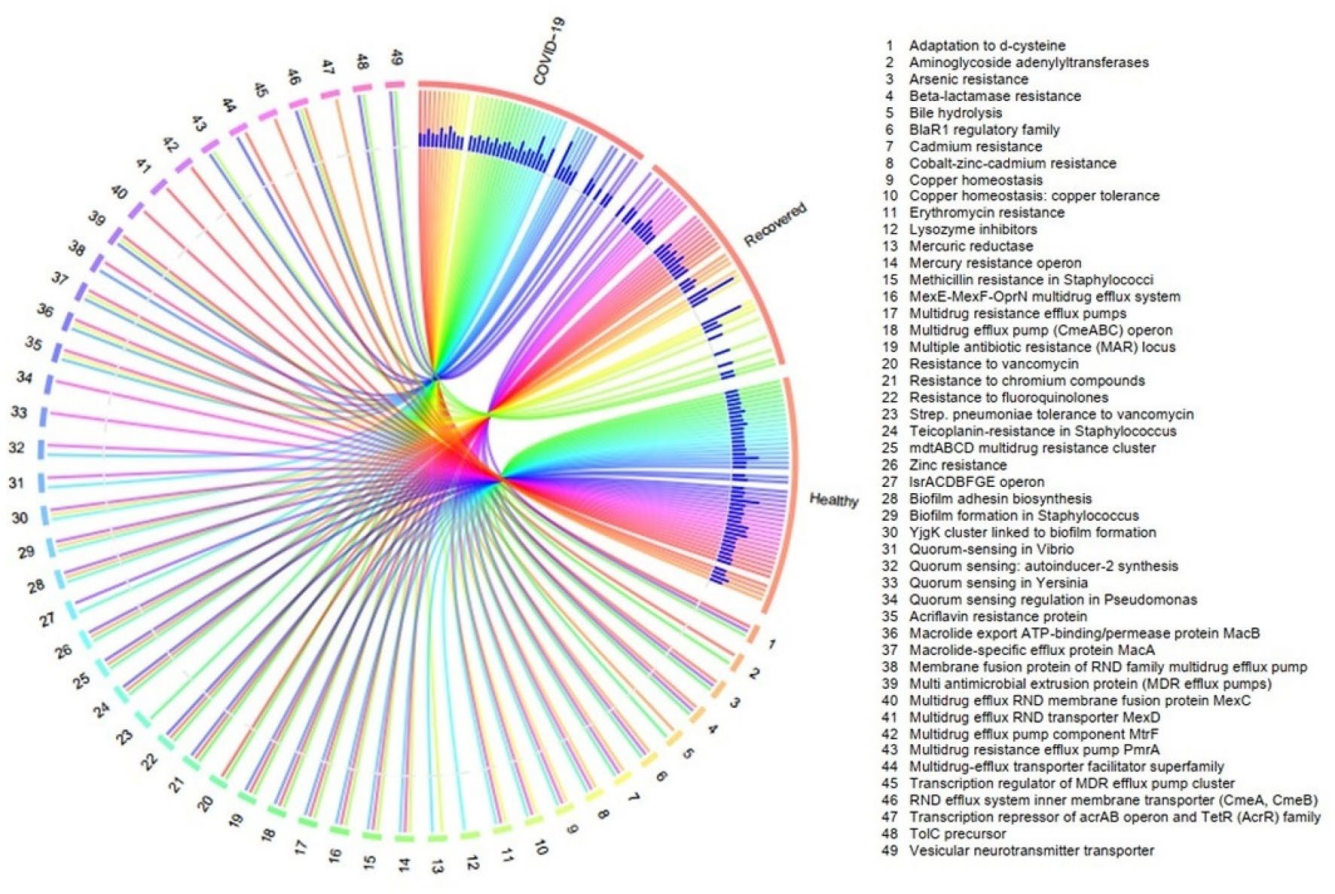
Distribution of the resistance to antibiotic and toxic compounds (RATC) genes in COVID-19, recovered and healthy control metagenomes. The circular plot illustrates the diversity and relative abundance of the RATC genes detected among the microbiomes of the three metagenomes through SEED subsystems analysis. The association of the RATC genes according to metagenome is shown by different colored ribbons and the relative abundances these genes are represented by inner blue colored bars. Some of the RATC functional groups shared among microbes of the three metagenomes (COVID-19, Recovered and Healthy), and rest are effectively undetected in the microbiomes of the other metagenomes. The numerical values 1–49 represent different RATC functional groups found across the study metagenomes. More data on RATC functional groups and their relative abundances are also available in Data S2. The circular plot is built using the OmicCircos (https://bioconductor.org/packages/release/bioc/html/OmicCircos.html), an R package.

In our current RNAseq data set, there was a broad variation in resistance to antibiotics and toxic compounds (RATCs) diversity and composition across three metagenomes (Fig. 8, Data S3). The categories and relative abundances of the RATCs were significantly correlated (p = 0.027, Kruskal–Wallis test) with the relative abundance of the associated microbiotas found in COVID-19, Recovered and Healthy control samples (Data S3). We detected 49 RATCs through MR analysis distributed in the microbial genomes of the three metagenomes (Fig. 8, Data S3). The COVID-19 associated microbiomes harbored the 40 different RATCs while 32 and 44 gene families were detected in the microbiomes of Recovered and Healthy controls. Of the detected RATC groups, genes associated with biofilm formation in *Staphylococcus* had the highest relative abundances in Recovered samples (80.0%) followed by COVID-19 (45.95%), and Healthy (3.57%) samples. Moreover, genes associated with acriflavin resistance were also predominantly abundant in COVID-19 (58.86%) and Recovered (66.67%) metagenomes. The other mostly abundant RATC functional groups in COVID-19 metagenome were quorum sensing: autoinducer-2 synthesis (29.73%), multidrug resistance cluster; *mdtABCD* (21.0%), cobalt–zinc–cadmium resistance (20.64%), BlaR1 regulatory family (15.56%), multidrug resistance efflux pump; *pmr*A (11.39%), resistance to fluoroquinolones (10.83%), *lsrACDBFGE* operon (10.81%) and multidrug resistance efflux pumps (10.25%) (Fig. 8, Data S3). Simultaneously, the nasopharyngeal microbiomes of the Recovered individual harbored higher abundance of genes encoding biofilm adhesin biosynthesis (20.0%), cobalt–zinc–cadmium resistance (18.97%), multiple antibiotic resistance (MAR) locus (17.50%), methicillin resistance in *Staphylococci* (14.66%), macrolide-specific efflux protein; *macA* (13.33%), arsenic resistance (12.93%), multidrug resistance efflux pumps (12.93%), and *YjgK* cluster linked to biofilm formation (11.0%) (Fig. 8, Data S3). Conversely, beta-lactamase resistance (35.71%), multidrug resistance efflux pump; *pmrA* (29.10%), biofilm formation in *Staphylococcus* (22.73%), quorum sensing in *Yersinia* (21.43%) and *Pseudomonas* (17.86%), and teicoplanin-resistance in *Staphylococcus* (12.82%) were top abundant RATC functional groups in the microbiomes of the Healthy controls. The rest of RATC functional groups also varied in their relative expression across the three metagenomes, being more prevalent in the COVID-19 and Recovered humans nasopharyngeal microbiomes (Fig. 8, Data S3).

**Figure 9:**
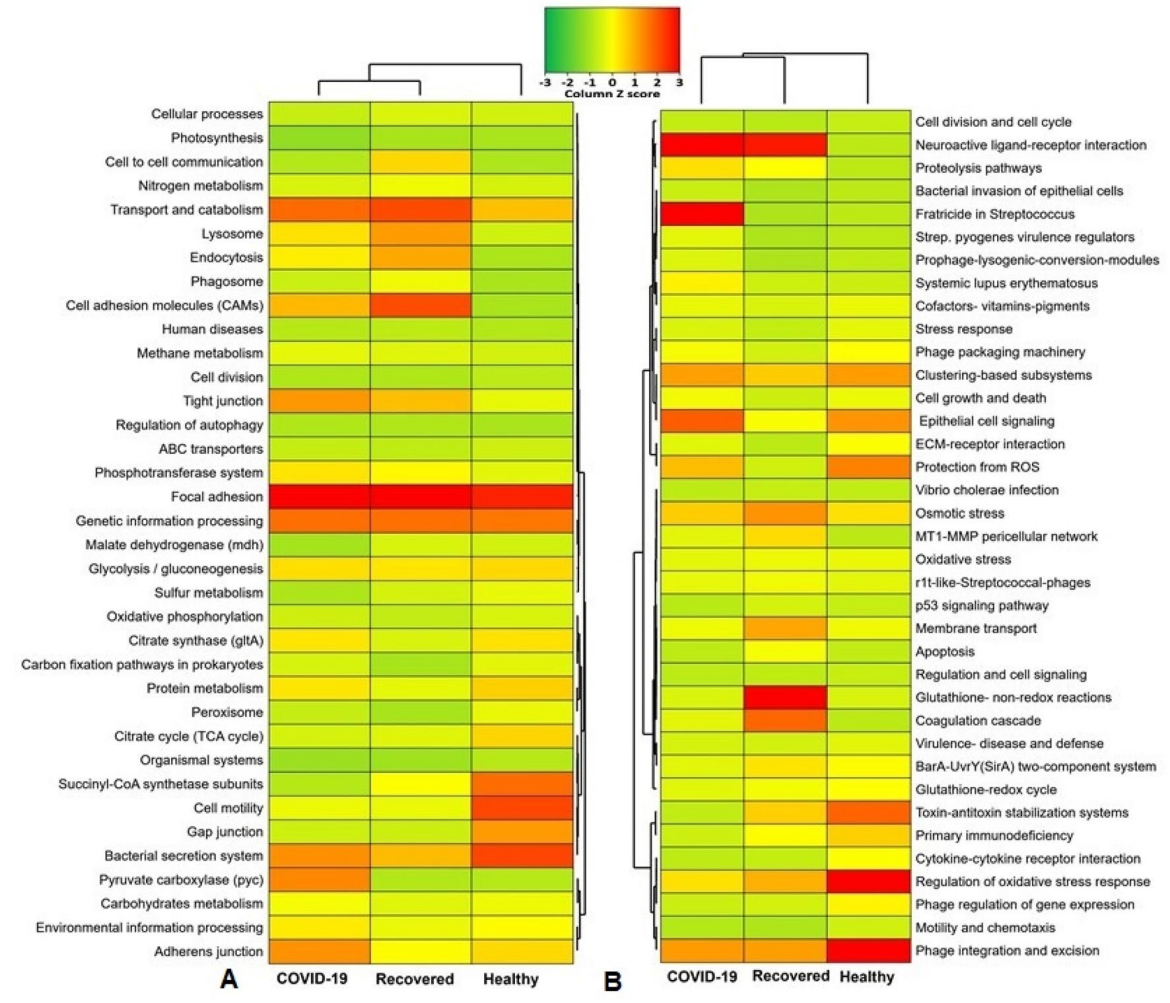
Functional annotation of the COVID-19, Recovered and Healthy nasopharyngeal sample related sequences. (**A**) Heatmap representing the average relative abundance hierarchical clustering of the predicted KEGG Orthologs (KOs) functional pathways of the microbiome across four metagenome groups. (**B**) Heatmap showing the average relative abundance hierarchical clustering of the predicted SEED functions in different levels among the microbiomes of four metagenomes. The color bars (column Z score) at the top represent the relative abundance of putative genes. The color codes indicate the presence and completeness of each KEGG and SEED module, expressed as a value between − 3 (lowest abundance) and 3 (highest abundance). The red color indicates the more abundant patterns, whilst green cells account for less abundant putative genes in that particular metagenome. The Heatmap is built through a stand-alone software tool called FunRich (http://www.funrich.org/).

By examining the correlation between the different gene families of the same KEGG pathway for COVID-19, Recovered and Healthy controls nasopharyngeal microbiomes, we found significant differences (p = 0.034, Kruskal–Wallis test) in their composition and relative abundances. Our analysis revealed that genes coding for pyruvate carboxylase (*pyc*) had several-fold overexpression in the microbiomes of the COVID-19 (26.31%) and Recovered (4.11%) groups compared to Healthy controls (1.80%) (Fig. 9A, Data S3). In addition, genes encoding for adherent junction (25.17%), tight junction (24.48%), environmental information processing (15.03%), carbohydrate metabolism (11.51%) and oxidative phosphorylation (7.26%) had higher relative abundances in the COVID-19 patient’s nasopharyngeal microbiomes (Fig. 9A, Data S3). The nasopharyngeal microbiomes of the Recovered people however had higher relative abundance of genes coding for focal adhesion (53.08%), transport and catabolism (40.44%), cell adhesion molecules (40.00%), genetic information and processing (35.12%), lysosome activity (28.68%), endocytosis (27.13%), and cell-to-cell communication (20.38%) (Fig. 9A, Data S3). Conversely, the gene families related to bacterial secretion system (40.09%), cell motility (39.83%), succinyl-CoA synthetase subunit C and D (34.35%), gap junction (27.27%), protein metabolism (19.49%), TCA cycle (18.72%) and citrate synthase (16.89%) remained overexpressed in Healthy humans nasopharyngeal microbiomes (Fig. 9A, Data S3).

We also sought to gain further insight into the SEED hierarchical protein functions represented by different genes, and found 37 statistically different (p = 0.013, Kruskal–Wallis test) SEED functions in COVID (COVID-19 and Recovered) and Healthy control metagenomes. Overall, the COVID-19 and Recovered samples associated microbiomes showed a higher relative abundance of these SEED functions compared to those of Healthy controls. For instance, cytokine–cytokine receptor interaction (50.0%), regulation of oxidative stress response (26.82%), phage integration and excision (24.92%), toxin–antitoxin regulation system (17.24%), protection from reactive oxygen species (14.96%), and phage regulation (6.06%) related genes had more than two-time overexpression in Healthy controls commensal nasopharyngeal microbiomes compared to COVID-19 and Recovered humans nasopharyngeal microbiomes (Fig. 9B, Data S3). The COVID-19 patients nasopharyngeal microbiomes were enriched in genes coding for cell growth and death (55.60%), *Streptococcus pyogenes* virulence regulators (42.44%), fratricide in *Streptococcus* (32.67%), neuroactive ligand receptor interaction (28.21%), epithelial cell signaling (20.35%), clustering-based subsystems (14.65%), proteolytic pathways (9.2%), prophage lysogenic conversion modules (3.69%) and bacterial invasion of epithelial cells (1.93%) compared to rest of the two metagenomes (Fig. 9B, Data S3). The Recovered humans nasopharyngeal microbiomes however had a higher abundance of SEED functions involved in glutathione: non-redox reactions (30.30%) and redox cycle (6.06%), coagulase cascade (20.0%), osmotic stress (16.07%), membrane transport (14.55%), MT1-MMP pericellular network (10.0%), BarA-UvrY(*SirA*) two-component regulatory system (9.29%), and oxidative stress (5.54%). In contrast, most of these SEED modules showed comparatively lower relative abundance in Healthy human nasopharyngeal microbiomes (Fig. 9B, Data S3).

## Discussion

SARS-CoV-2 infection may predispose to secondary microbial infection which is associated with poor clinical outcome especially among critically ill patients. Therefore, the complex crosstalk between SARS-CoV-2 and commensal microbes of different body systems is essential for the functioning of the immune system. In the present study, we demonstrated a remarkable shift in the diversity and composition of the nasopharyngeal microbiomes (bacteria, viruses and archaea) of COVID-19 and Recovered people compared to the Healthy humans through the state-of-the-art RNAseq technology. The findings of the present study revealed that SARS-CoV-2 infection reduces commensal bacteria with inclusion of pathobionts in the nasopharyngeal tract of human (Fig. 1). Furthermore, we identified a number of microbial genomic features, altered metabolic pathways, and functional genes associated with COVID-19 pathogenesis. Although the dysbiosis of human gut microbiome by the infection of SARS-CoV-2 has been reported in several earlier studies^8,21,22^, this study for the first time determined the interactions and consequences of SARS-CoV-2 infection with resident nasopharyngeal microbiome of human.

Since some of the COVID-19 patients showed persistent symptoms after recovery and/or subsequently develop multisystem inflammation^8^, we hypothesized that the altered nasopharyngeal microbiomes during SARS-CoV-2 infections could lead to abnormal inflammatory reactions to worsen the symptoms and treatment outcomes of COVID-19. The microbiome diversity (alpha and beta diversity) measures suggest that microbial dysbiosis is closely linked to SARS-CoV-2 infections. Our analysis also showed a substantial microbial disparity between COVID-19 and Healthy-controls nasal samples keeping the closest relationship between COVID-19 and Recovered people’s nasopharyngeal samples, and related microbial signatures. The COVID-19-patients nasopharyngeal microbiomes had significantly lower variance in diversity than those of healthy humans, agreeing with several recent studies^23,24^. In a recent study, Zou et al. reported that loss of salutary species in COVID-19 pathogenesis in most patients, despite the clearance of SARS-CoV-2 virus, is associated with a more long-lasting detrimental effect to the gut microbiome^24^. The nasopharyngeal microbiomes predominantly identified in this study are bacteria (> 81. 0%), however, other concomitant microbial players like viruses and archaea were also detected, highlighting the novel insights of diverse microbial association with SARS-CoV-2 to exacerbating the course, common symptoms and treatment outcomes of the COVID-19^22,25,26^. In COVID-19 samples, the relative abundances of *Firmicutes*, *Bacteroidetes* and *Proteobacteria* phyla, and their related genera such as *Streptococcus*, *Veillonella*, *Prevotella*, *Acinetobacter*, *Staphylococcus*, *Clostridium*, *Coprobacillus*, and *Neisseria* remained much higher compared to those of Recovered and Healthy samples (Table 1). For instance, *Streptococcus* and *Veillonella* had more than five-fold higher relative abundances in COVID-19 samples than the Recovered and Healthy samples. *Prevotella*, *Veillonella*, and *Streptococcus* are the most common genera that reside in the lungs and nasopharynx^27,28^, and recently these genera have been reported to play an opportunistic role in the progression of lung infections^28,29^. Despite having almost homogeneous genetic backgrounds and living in the same region, there were vast differences in microbiome signatures in nasal cavities of Healthy controls, COVID-19 patients and Recovered individuals. The Healthy people had higher number of bacterial genera compared to COVID-19 patients and Recovered individuals. Moreover, the inter-individual microbiome signature (bacterial genera) of participants was also observed, and was largely stable in COVID-19 patients. Notably, COVID-19 patients mainly had increased numbers of opportunistic pathogens, a part of commensal microbiota that may become pathogenic in the event of host perturbation, such as dysbiosis or immunocompromised host. The human microbiota shows a remarkable amount of diversity among different individuals, and there are ample of emerging evidences that the microbial communities can be altered to change pathophysiological state or even cure of disease is a compelling drivers of microbiome variation^30^. Moreover, co-infections in COVID-19 patients could also be attributed by different variants and strains of SARS-COV-2^5,31^.

The attributes of microbiomes, for instance, pathogenicity, virulence, antibiotic resistance, and metabolic potentials are linked with the species or strain specific genomic characteristics^22,23,32^. We demonstrated that the Healthy samples possessed highest number of commensal bacterial species while SARS-CoV-2 infections and subsequent therapy reduced 37.78% and 54.23% bacterial species in COVID-19 patients and Recovered humans nasopharyngeal samples, respectively indicating the perturbations of beneficial microbes amid COVID-19. The COVID-19 patients and Recovered people had sole association of 50.16% and 29.04% bacterial species, respectively. These results are in line with several previous studies who reported that cross-talk and/or interactions between SARS-CoV-2 and oral microbiomes^26^, and SARS-CoV-2 and gut microbiomes^33^ is associated with the pathophysiology of lung diseases. Therefore, majority of the bacterial species identified in COVID-19 (66.26%) and Recovered (76.78%) samples had an opportunistic inclusion by the depletion of beneficial commensal microbes during the course of SARS-CoV-2 pathogenesis and recovery phase. The composition of the bacterial communities in the nasopharynx is more diverse than any other parts of the upper respiratory tract, which are characterized by different *Streptococcal* species, *Staphylococcus* spp., *Haemophilus* spp., *Neisseria* spp., *Rothia* spp., and anaerobes, including *Veillonella* spp., *Prevotella* spp., and *Leptotrichia* spp.^18,34,35^. Despite some preliminary evidence, it is still unclear whether similar synergies exist between SARS-CoV-2 and specific bacterial infections as exist with influenza^36,37^. Surprisingly, 68.43% bacterial species had sole association with Healthy nasopharyngeal samples, which were not detected in COVID-19 patients and Recovered humans indicating the potential dysbiosis of commensal microbiomes during pathophysiology of SARS-CoV-2 infections. In this study, different species of *Pedobacter*, *Sphingobacterium*, *Pseudomonas*, *Enterobacter*, and *Flavobacterium* genera remained top abundant in Healthy human nasopharyngeal swabs. These microbes are ubiquitous and isolated from natural environments, such as soil and water, readily cleared by host defenses and rarely involved in human infection^10,18,34,38^. Though, the nature of nasopharyngeal commensal bacteria that exert antiviral effect in the lungs is still elusive, some members of these commensal species (desaminotyrosine producing anaerobes) are important in the priming of the pulmonary innate immune system^22^. Despite the beneficial role of these commensal bacterial species are largely unknown, reduction in the composition and relative abundances of these commensal bacteria from the nasopharyngeal tract, play an important role in microbiome formation, which can significantly affect the immune response against SARS-CoV-2 infections^39^.

The cutting-edge RNAseq approach of this study also provided an exciting opportunity for investigating the integrated cross-kingdom interactions of ‘multibiome’ such as viruses (other than SARS-CoV-2) and archaea which contributed about 20.0% of the total microbial sequence reads. Unlike bacteria, the diversity and composition of viruses and archaea always remained much lower in both COVID-19 and Healthy samples. In this study, of the detected viral reads (18.0%), the *Alphacoronvirus* (HCoV-NL63) and *Betacoronovirus* (SARS-CoV-2 and Pangolin coronavirus) comprises majority (> 85%) sequences indicating that both Alpha- and Beta-coronaviruses are simultaneously prevailing in Bangladesh. SARS-CoV-2 was the predominantly abundant (> 99.0% relative abundances) viral strain in COVID-19 and Healthy controls (84%) metagenomes, and rest of the viral strains concurrently detected with SARS-CoV-2 were belonged to *Alphacoronavirus* and *Siphoviruses.* On the contrary, HCoV-NL63 was the most abundant viral strain (90.89%) in Recovered humans’ nasopharyngeal samples followed by SARS-CoV-2 (2.76%). Human coronaviruses (HCoVs) have only recently been shown to cause both lower and upper respiratory tract infections. The endemic HCoV-NL63 can cause co-infections, sequential infections or can be co-detected with each other or with other respiratory viruses like SARS-CoV-2^40,41^. Recent studies reported that infection with HCoV-NL63 is common worldwide, and associated with many clinical symptoms in the immunocompromised SARS-CoV-2 patients^41,42^. Other viral strains identified in the samples of COVID-19, Recovered and Healthy metagenomes were mostly represented by different strains of bacterial phages. Recently, stronger relationship among SARS-CoV-2, bacteria, fungi, and other viruses have been reported from different countries^43–45^. The respiratory tract microbiomes support large populations of viruses, mostly bacteriophages (phages) that infect specific bacterial species, and can appear as free phage virions (phage particles) or as dormant prophages. Since most secondary bacterial infections occur in immunocompromised or immunodeficient SARS-CoV-2 patients, phages can induce immune responses these patients^46^. The archaeal fraction of the human nasopharyngeal microbiomes across three metagenomes were predominated by different methanogenic and thermophilic genera. Though the role of these accompanying microbiomes in the pathophysiology of COVID-19 remains a mystery, it may include virus-induced airway damage, cell loss, goblet cell hyperplasia, altered mucus secretion, reduced ciliary beat frequency, function and clearance, reduced oxygen exchange, and damage to the immune system^47^.

With advancement in metagenomic sequencing techniques and downstream bioinformatics analyses, it is now possible to critically examine and analyze various microbial communities and their concurrent antimicrobial resistance in different body sites including the NT. One of the important findings of the present study is the concurrent detection of various homologs of RATCs belonging to different protein families among the microbiome of three metagenomes. Interestingly, the composition and relative abundance of the RATCs detected in this study remained significantly correlated with microbiome signature and their relative abundances in the nasal cavities of the Healthy humans, COVID-19 patients and Recovered individuals. The observed variations in the composition and relative expression of RATCs in COVID-19, Recovered and Healthy controls corroborates with the dynamic dysbiosis of microbiomes in the corresponding metagenomes, their genetic diversity, and selective pressure for the maintenance of antimicrobial resistance. The microbiomes of COVID-19 patients and Recovered humans nasopharyngeal swabs were enriched with genes coding for biofilm formation in *Staphylococcus*, quorum sensing: autoinducer-2 synthesis, *mdtABCD*, cobalt–zinc–cadmium, acriflavine, arsenic, fluoroquinolones and methicillin resistance, BlaR1 regulatory family, *mac*A, MAR locus, *pmr*A, *YjgK* cluster, and *lsrACDBFGE* operon. Conversely, beta-lactamase resistance, quorum sensing in *Yersinia* and *Pseudomonas*, and teicoplanin-resistance in *Staphylococcus* encoding genes remained predominantly abundant in nasopharyngeal microbiomes of the Healthy controls. Remarkably, since the beginning of the COVID-19 pandemic, there has been growing concern for a potential rise in AMR secondary to increased antibiotic prescription for COVID-19 patients^48^. Moreover, severe COVID-19, which particularly affects elderly patients with multiple comorbidities, may be an important factor in determining changes in colonization pressure and multidrug resistance^49^.

The functional analysis of the microbiomes revealed that genes coding for pyruvate carboxylase (*pyc*), adherent junction, tight junction, environmental information processing, carbohydrates metabolism, and oxidative phosphorylation had higher relative abundances in the COVID-19 patients nasopharyngeal microbiomes compared to those of Recovered and Healthy control microbiomes. These metabolic functional changes further lead to increased cytokine–cytokine receptor interaction, regulation of oxidative stress response, phage integration and excision, toxin–antitoxin regulation, protection from reactive oxygen species, and phage regulated gene expression in COVID-19 associated microbiomes as also reported previously in other viral diseases^50,51^. The Recovered humans NT microbiomes were enriched with genes coding for focal adhesion, transport and catabolism, cell adhesion molecules, genetic information and processing, lysosome activity, endocytosis, cell-to-cell communication and glycolysis. The pertinent genomic potentials of the COVID-19 patient and Recovered humans nasopharyngeal microbiomes were also evidenced by the higher expression of genes involved in glutathione: non-redox reactions, redox cycle, coagulase cascade, osmotic stress, membrane transport, MT1-MMP pericellular network, and BarA-UvrY (*SirA*) two-component regulatory activities. Conversely, the Healthy people nasopharyngeal microbiomes showed a lower abundance and expression of these metabolic functional genes. The metabolic health of an individual is represented by the proper functioning of organismal metabolic processes coordinated by multiple physiological systems^51^. The differentially abundant functions and pathways identified in this study corroborates with the findings from previous reports, relating to decreased lipid and glycan metabolism, increased carbohydrate metabolism, and other characteristics of the microbiome linked to COVID-19^52^. Several predicted functional pathways differed between COVID-19 and Healthy controls, perhaps reflecting metabolic changes associated with the progression of COVID-19 pathogenesis, and novel host–microbiome interactions in SARS-CoV-2 infected patients.

## Conclusions

Host–microbiomes interactions are exceptionally complex especially in SARS-CoV-2 infections. Our RNAsSeq metagenomic analyses revealed that SARS-CoV-2 infection had significant effect on the diversity and composition of nasopharyngeal microbiomes of human. The identifiable changes in the microbiome diversity, composition and associated genomic features demonstrated in this study might be associated with the development, treatment, and resolution of COVID-19. The SARS-CoV-2 infection results in remarkable depletion of nasopharyngeal commensal microbiomes of Healthy humans with inclusion of different opportunistic pathogens in COVID-19 and Recovered samples. Several predicted functional pathways differed between COVID-19 patient and Recovered people nasopharyngeal samples compared to healthy individuals which reflect the roles microbial metabolic changes with the progression of SARS-CoV-2 pathogenesis. These interactions are further complicated by the common co-existence of microbiota that interact with both host and SARS-CoV-2. Therefore, findings of this study may serve as a benchmark for microbiome-based diagnostic markers and therapeutics for this pandemic disease. However, future time-course studies with a larger sample size are needed to elucidate the dynamic changes in composition and diversity of commensal microbiomes, and the inclusion of pathobionts in the whole respiratory system and gut during the progression of COVID-19.

## Methods

### Subject recruitment and sample collection

This study was conducted in accordance with the guidelines of the Director General of Health Services (DGHS) of Bangladesh during May to July, 2020. The study participants provided written informed consent consistent with the experiment. Twenty-two (n = 22) nasopharyngeal samples (including COVID-19 = 8, Recovered = 7, and Healthy = 7) were collected from Dhaka city of Bangladesh. The patients were diagnosed positive for COVID-19 on an average 4.7 days (range 2–9 days) after the onset of clinical signs (SARS-CoV-2 positive through RT-qPCR). On an average, 17.5 days (range 11–32 days) after the first confirmation of SARS-CoV-2 infection these patients were tested negative for COVID-19 and categorized as recovered people (Table S1). The average age of the study people was 41.86 (range 22–72) years, and of them, 68.18% and 38.82% were male and females, respectively (Data S1). The RT-qPCR was performed for *ORF1ab* and *N* genes of SARS-CoV-2 using novel Coronavirus (2019-nCoV) Nucleic Acid Diagnostic Kit (PCR-Fluorescence Probing, Sansure Biotech Inc.) according to the manufacturer’s instructions. Viral RNA was extracted using a PureLink viral RNA/DNA minikit (Thermo Fisher Scientific, USA). Thermal cycling was performed at 50 °C for 30 min for reverse transcription, followed by 95 °C for 1 min, and then 45 cycles of 95 °C for 15 s, 60 °C for 30 s on an Analytik-Jena qTOWER instrument (Analytik Jena, Germany).

Nasopharyngeal samples from COVID-19 and Recovered subjects were collected on the test day (day of COVID-19 positive and COVID-19 negative identification), and subsequently sent for RNA extraction and sequencing. The Healthy control subjects were randomly selected and these people did not show any signs and symptoms of respiratory illness, and nasopharyngeal swabs from these Healthy people were collected following the same protocol for COVID-19 and Recovered humans, and immediately sent for RNA extraction and sequencing.

### RNA sequencing

We utilized total RNAseq approach for the metagenomics component of the study. The cDNA of all 22 samples was used to prepare paired-end libraries with the Nextera DNA Flex library preparation kit (Illumina, Inc., San Diego, CA) according to the manufacturer’s instructions. Paired-end (2 × 150 bp reads) sequencing of the prepared library pool of the samples was performed using a NextSeq high throughput kit with an Illumina NextSeq 550 sequencer at the Genomic Research Laboratory, Bangladesh Council of Scientific and Industrial Research (BCSIR), Dhaka, Bangladesh.

### Taxonomic mapping, classification, diversity and community analysis

The paired-end sequences of COVID-19, recovered and healthy samples (n = 22) were analyzed using both mapping-based and assembly-based hybrid methods of IDseq^53^ and MG-RAST (release version 4.1) (MR)^54^. IDseq- an open-source cloud-based pipeline has been used to assign taxonomy with NT L (nucleotide alignment length in bp) >= 50 and NT %id >= 97. This pipeline used quality control, host filtering, assembly based alignment and taxonomic reporting aligning to NCBI nt (nucleotide database). The sequencing reads were filtered through BBDuk (with options k = 21, mink = 6, ktrim = r, ftm = 5, qtrim = rl, trimq = 20, minlen = 30, overwrite = true) to remove Illumina adapters, known Illumina artifacts, and phiX. Any sequence below these thresholds or reads containing more than one ‘N’ were discarded^32^. In the current study, 120.50 million reads were generated from the samples of three metagenomes with an average of 5.0 million reads per sample passed the quality control steps, and of these quality reads, an average of 3.22 million aligned reads per sample mapped to the ribosomal (rRNA) reference gene libraries (Data S1). Alpha diversity (diversity within samples) was estimated using the Shannon and Simpson diversity indices. To visualize differences in bacterial diversity, a PCoA (at genus level) was performed based on the Bray–Curtis distance method. Microbial taxa that were detected in one group of sample but not detected in rest of the two groups are denoted as solely (unique) associated microbiomes^32^.

### Functional profiling of the microbiomes

We performed the taxonomic functional classification through mapping the reads onto the Kyoto Encyclopedia of Genes and Genomes (KEGG) database^55^, and SEED subsystem identifiers^54^ on the MR server using the partially modified set parameters (*e-*value cutoff: 1 × 10^–30^, min. % identity cutoff: 60%, and min. alignment length cutoff: 20)^56^.

### Statistical analysis

The non-parametric test Kruskal–Wallis rank sum test was used to evaluate differences in the relative percent abundance of taxa in COVID-19, Recovered and Healthy sequence data. The gene counts were normalized by dividing the number of gene hits to individual taxa/function by total number of gene hits in each metagenome to remove bias due to differences in sequencing efforts. In addition, statistical tests were also applied with non-parametric test Kruskal–Wallis rank sum test at different KEGG and SEED subsystems levels through IBM SPSS (SPSS, Version 23.0, IBM Corp., NY, USA)^32^.

### Ethical statement

The protocol for sample collection from COVID-19, recovered and healthy humans, sample processing, transport, and RNA extraction was approved by the National Institute of Laboratory Medicine and Referral Center of Bangladesh.

## Supplementary Information

Supplementary Information 1.

Supplementary Information 2.

Supplementary Information 3.

Supplementary Information 4.

Supplementary Information 5.

## Data Availability

All data needed to evaluate the conclusions in the paper are present in the paper and/or the Supplementary Materials. The sequence data reported in this article has been deposited in the National Center for Biotechnology Information (NCBI) under BioProject accession number PRJNA720904.
