# Supplementary material for: SARS-CoV-2 infection reduces human nasopharyngeal commensal microbiome with inclusion of pathobionts
Source: Sci Rep. 2021 Dec 15;11:24042. doi: 10.1038/s41598-021-03245-4 (PMC8674272; doi:10.1038/s41598-021-03245-4)
Supplement: Supplementary file 1 — Supplementary Information 1. [file 41598_2021_3245_MOESM1_ESM.docx]

**Table S1.** Clinical diagnosis, treatment and recovery history of the study population.

| **Sample_ID** | **Age** | **Gender** | **COVID-19 test**  **(Days after onset of symptoms)** | **Recovered***  **(Days after COVID-19 +Ve test)** |
| --- | --- | --- | --- | --- |
| COVID-1 | 22 | M | 3 | 14 |
| COVID-2 | 35 | F | 5 | 12 |
| COVID-3 | 45 | M | 1 | 11 |
| COVID-4 | 39 | M | 5 | 14 |
| COVID-5 | 32 | M | 5 | 21 |
| COVID-6 | 34 | M | 9 | 32 |
| COVID-7 | 38 | F | 4 | 14 |
| RECOV-1 | 38 | F | 2 | 14 |
| RECOV-2 | 34 | M | 9 | 32 |
| RECOV-3 | 22 | M | 5 | 17 |
| RECOV-4 | 35 | F | 3 | 12 |
| RECOV-5 | 45 | M | 4 | 17 |
| RECOV-6 | 32 | M | 7 | 21 |
| RECOV-7 | 39 | M | 4 | 14 |
| Average | 35 |  | 4.72 | 17.5 |

* The Recovered people became COVID-19 tests negative.
