# Supplementary material for: SARS-CoV-2 infection reduces human nasopharyngeal commensal microbiome with inclusion of pathobionts
Source: Sci Rep. 2021 Dec 15;11:24042. doi: 10.1038/s41598-021-03245-4 (PMC8674272; doi:10.1038/s41598-021-03245-4)
Supplement: Supplementary file 2 — Supplementary Information 2. [file 41598_2021_3245_MOESM2_ESM.docx]

**Table S2.** Taxonomic distribution of bacteria, viruses and archaea in COVID-19, Recovered and Healthy individual metagenomes.

| **Taxonomic Rank** | **Bacteria** | | |
| --- | --- | --- | --- |
|  | **Healthy** | **COVID-19** | **Recovered** |
| Phyla (n=28) | 28 | 27 | 23 |
| Order (n=93) | 92 | 89 | 79 |
| Family (n=210) | 204 | 198 | 181 |
| Genus (n=532) | 509 | 486 | 421 |
| Species (n=2281) | 1476 | 919 | 675 |
| **Taxonomic Rank** | Virus | | |
|  | **Healthy** | **COVID-19** | **Recovered** |
| Order (n=7) | 4 | 7 | 5 |
| Family (n=26) | 9 | 19 | 16 |
| Genus (n=53) | 22 | 35 | 31 |
|  | | | |
| **Taxonomic Rank** | **Archaea** | | |
|  | **Healthy** | **COVID-19** | **Recovered** |
| Order (n=14) | 12 | 14 | 8 |
| Family (n=20) | 16 | 20 | 8 |
| Genus (n=48) | 35 | 42 | 20 |
