# Supplementary figures and images for: SARS-CoV-2 infection reduces human nasopharyngeal commensal microbiome with inclusion of pathobionts
Source: Sci Rep. 2021 Dec 15;11:24042. doi: 10.1038/s41598-021-03245-4 (PMC8674272; doi:10.1038/s41598-021-03245-4)

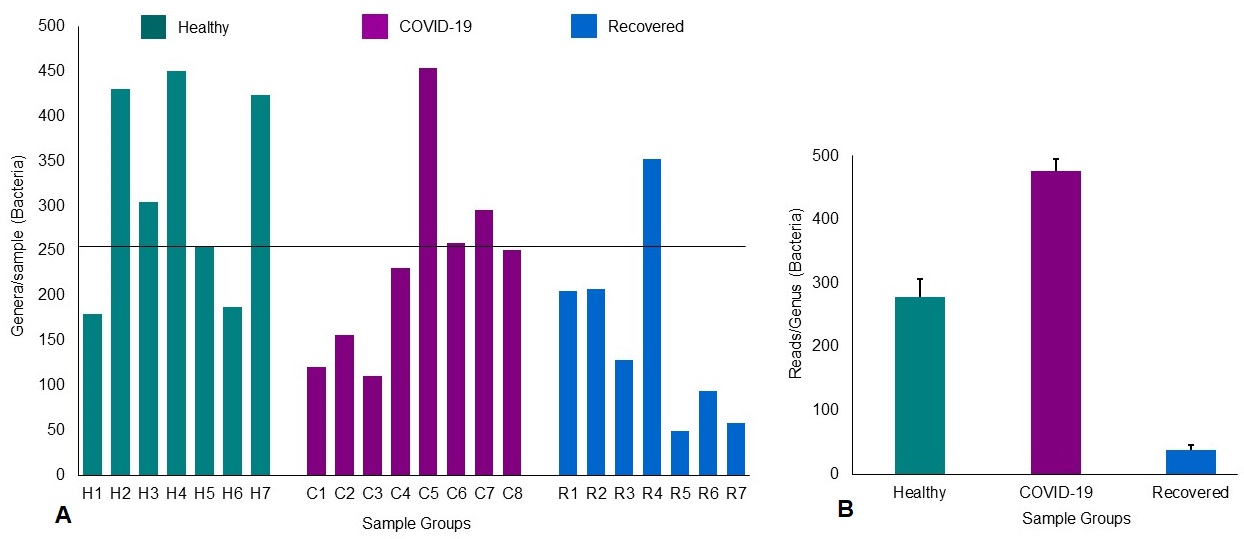

Supplement: Supplementary file 3 — Supplementary Information 3. [file 41598_2021_3245_MOESM3_ESM.jpg]
